# Countdown to 2030: overview of current and planned health financing reforms for universal health coverage in the WHO African Region

**DOI:** 10.7189/jogh.15.04233

**Published:** 2025-08-08

**Authors:** Doris Osei Afriyie, Diane Karenzi Muhongerwa, Juliet Nabyonga-Orem, Ogochukwu Chukwujekwu

**Affiliations:** 1 World Health Organization Regional Office for Africa, Congo Brazzaville, Congo; 2 World Health Organization, Country Office Namibia, Namibia; 3 Centre for Health Professions Education, Faculty of Health Sciences, NorthWest University, Potchefstroom, South Africa

## Abstract

**Background:**

Countries in the World Health Organization (WHO) African region are lagging behind in the global push toward universal health coverage (UHC), a core component of the 2030 Agenda for Sustainable Development. As the target year steadily approaches, it is crucial to assess countries’ current and planned health financing reforms to understand their path towards UHC. We examine both current and planned health financing reforms in the WHO African region and assess their implications for UHC.

**Methods:**

We sent a survey to all 47 Member States of the WHO African Region in August 2024 about their current and planned health financing reforms in the three health financing functions of revenue raising, pooling, and purchasing, as well as public finance management. We used responses from 43 countries, of which 18 countries had a current endorsed national health financing strategy to assess the implications of their reforms based on current literature on using health financing to progress towards UHC goals.

**Results:**

Of the 43 countries in the WHO African Region that responded to our survey, 33 (77%) have current or planned health financing reforms across the various health financing functions. A major focus of these reforms is on establishing contributory health insurance schemes, despite their limited potential to address the region’s challenges. Additionally, countries are prioritising expanding performance-based financing and provider payment mechanisms. These purchasing strategies could improve service coverage and quality of care if implemented within robust public finance management structures.

**Conclusions:**

Countries in the WHO African Region are adopting and planning various health financing reforms to achieve UHC. To ensure success, they will require support in effectively implementing evidence-based reforms in the areas of purchasing and reducing fragmentation from various coverage schemes.

The 2023 Global Monitoring Report on Universal Health Coverage (UHC) highlighted the slow progress of the World Health Organization (WHO) African Region towards UHC [1]. In 2021, the region’s population-weighted UHC service coverage index score was 44, nearly double its score in 2000, yet still the lowest among all WHO regions and significantly below the global average of 68. Growth has stagnated since 2015, with the COVID-19 pandemic further exacerbating the decline in the coverage of essential health services, hindering progress towards UHC. Persistent inequalities in access to essential health services remain a major challenge both within and across these countries [2]. Additionally, the financial hardship is growing, as the share of households facing out-of-pocket health spending exceeding 10% of their household increased from 8.1% in 2017 to 8.6% in 2019 [1].

Health financing is a critical building block of health systems in ensuring progress towards UHC, a key component of the Sustainable Development Goal (SDGs). Core health financing functions such as revenue raising can directly impact UHC by improving service coverage and financial protection [3]. For example, increasing public revenue allocated to health enhances pre-payment funding, which subsequently strengthens financial protection and support service utilisation goals [4].

Most countries in the WHO African Region face significant challenges due to low levels of domestic public funding for health. In 2022, only four countries met the Abuja target of allocating at least 15% of government budgets to health [5]. The median public health expenditure in the region was approximately USD 14 per capita [5], which is substantially lower than the estimated USD 90–112 per capita required to support health systems in expanding service coverage in low-income countries [6]. Due to low public health spending, out-of-pocket expenditure is a major source of health funding in many countries across the region; in 2022, it accounted for 50% of current health expenditure in 11 countries [5]. Furthermore, external funding remains a critical source of support for health systems in the region, yet is highly unpredictable. Prior to the COVID-19 pandemic, development assistance for health remained largely stagnant, with noticeable increases observed only in 2020 and 2021 [7].

To move closer to UHC, the WHO has urged countries to assess their health financing policies and implement the necessary actions to enhance equity in service utilisation and financial protection [8]. In response, over the past decade, many low- and middle-income countries have implemented various health financing reforms to support progress towards UHC. These reforms have included changes to revenue collection, resource pooling, and purchasing of health services, such as provider payment mechanisms and contracting models.

With only a few years remaining until 2030 – the endpoint year for the SDGs – it is essential to examine the health financing reforms countries have adopted for UHC and identify the needed steps to accelerate progress. Previous research on health financing reforms, while extensive, has primarily focussed on individual schemes or specific health financing functions such as revenue raising or purchasing [9,10]. As all health financing functions are critical for UHC, it is important to have a system-wide perspective of reforms countries are prioritising to progress towards UHC. Moreover, earlier analyses have concentrated on a few countries within the region and did not specify actions countries are taking for UHC [11].

To address this gap, we examined the current and planned health financing reforms in the WHO African Region using the WHO conceptual framework for health financing for UHC [4]. We also drew from published literature to assess the implications of these reforms on increasing service coverage and financial protection. We analysed survey responses from 43 out of the 47 Member States about their current and planned health financing reforms. Additionally, we reviewed the national health financing policy/strategy documents from all 18 countries with an endorsed national health financing strategy.

## METHODS

### Conceptual framework

We adapted WHO’s framework for health financing for UHC to examine countries’ reforms [4]. This framework shows the three core functions of health financing, which include revenue raising, pooling, and purchasing of health services along with benefit design (Figure S1 in the **Online Supplementary Document**). We drew from the work of Asante and colleagues [12] who demonstrated the relevance of this framework in assessing health financing systems in sub-Saharan Africa.

Revenue raising is essential for mobilising adequate funds for health. To progress towards UHC intermediate objectives and goals, the WHO recommends three key principles for revenue-raising policy [13]. First, shifting from out-of-pocket payments to a predominant reliance on public funding through compulsory and pre-paid mechanisms can increase efficiency. Second, ensuring a stable and predictable flow of funds is crucial for efficiency. Finally, ensuring fairness in the way revenues are raised can promote equity in resource distribution.

Pooling is a core health financing function that utilises prepaid funds to spread the financial risk associated with the use and cost of health services across individuals with varying risk levels [14]. This risk is managed through the redistribution of funds from healthier pools to those with higher health needs. The WHO outlines four key principles for reforms in pooling to increase redistributive capacity and efficiency [15]. The first is making coverage compulsory or automatic to increase the size and diversity of the pool. The second is merging different pools to reduce fragmentation and inefficiencies in service delivery. The third is cross-subsidising through risk adjustment, whereby funds allocated to pools are based on the relative health risks of their populations. Finally, harmonising pools in a complementary manner, such as aligning provider payments and benefit packages [15]. In the WHO African region, fragmentation of health coverage schemes remains a significant issue in many countries. For instance, Senegal operates at least five distinct health coverage schemes, each targeting specific populations. These countries rely on various funding schemes, such as government budgets, targeted population schemes by donors and social health insurance (SHI) schemes [16]. However, the limited coordination among these fragmented pools ultimately hampers efficiency and leads to inequitable resource distribution [17].

Purchasing health services is a core function of health financing that involves allocating funds to providers for the health services they deliver [18]. To advance towards UHC, countries have been encouraged to implement strategic purchasing which involves using information on provider performance and population health needs to guide funding and create incentives. The WHO has four core areas and policy questions of strategic purchasing: specifying services and interventions, selecting providers, designing financial and non-financial incentives such as provider payment mechanisms, and contracting agreements [18]. In the WHO African Region, strategic purchasing arrangements is an issue as government health budgets in many countries typically do purchase services without effective use of strategic levers [19]. Additionally, providers have limited managerial and financial autonomy in decision spaces to shift funds across line-item budgets.

We also included public financial management (PFM), as public funding constitutes a major share of financing of health in many countries, and as a robust PFM system is crucial for advancing towards UHC [20]. PFM encompasses the rules and processes that govern how budgets are formulated, allocated, utilised, and monitored. A robust PFM system enables predictability and adequacy in budget, alignment of allocations with sector priorities, and better budget execution in a flexible manner [20,21]. It also influences efficiency, transparency, and accountability, all of which are crucial for UHC goals. Based on countries’ experiences and the literature, the WHO recommends four actions, particularly in the WHO Africa Region, to align PFM with UHC objectives [20,21]:

1. Increase understanding of public budgeting roles, processes, and practices among health sector stakeholders.

2. Actively contribute to PFM reform design and implementation of PFM reforms related to health.

3. Shift from input-based budgeting to goal-oriented, programmatic health budgets.

4. Lead policy design for health-specific PFM interventions, including financial autonomy of health facilities.

We employed multiple methods to explore planned and ongoing health financing reforms to accelerate progress towards UHC. In August 2024, we sent a survey on health financing reforms to all 47 Member States in the region. The respondents were health financing focal points in WHO country offices who work closely with their respective ministry of health and participate in their country’s technical working group on health financing. The survey (Table S1 in the **Online Supplementary Document**) included questions on the status of countries’ national health financing strategy documents, as well as ongoing and planned health financing reforms.

Through the survey, we identified countries that have endorsed and are implementing a current health financing strategy in 2024. A national health financing strategy focusses on the entire health system rather than a specific population or individual scheme within the system [8]. It consists of diagnosing the country’s health system performance relative to its goals and determining the priority actions and policies needed in revenue raising, pooling, purchasing, governance, and capacity building to implement reforms. To complement the survey responses, we conducted a document review of the 18 countries with a current and endorsed national health financing strategy (Table S2 in the **Online Supplementary Document**). This review involved analysing the specific strategies outlined in their documents for revenue raising, pooling, purchasing, and public finance management. Additionally, we examined the major activities they planned to undertake for the implementation of these strategies.

We analysed the survey responses using descriptive statistics, summarising the reforms across the three health financing functions-revenue raising, pooling, and purchasing, as well as public finance management using frequencies and percentages. To ensure consistency and gain insights into their reforms, we triangulated the survey data with information from the health financing strategy documents. We validated our findings by sharing with WHO country health financing focal points. We also presented the findings in a WHO health financing points’ meeting, where minor considerations were raised and incorporated into the findings.

## RESULTS

### Health financing reforms in the African Region

Forty-three out of the 47 countries responded to the survey, giving a response rate of 91%. Thirty-three (76.74%) of these countries have ongoing or planned health financing reforms (Table S3 in the **Online Supplementary Document**). These reforms span various health financing functions, with the majority focussing on revenue raising and purchasing mostly through contributory health insurance schemes and performance-based financing (**Table 1**).

**Table 1:** Overview of current and planned health financing reforms in the WHO African Region

Health financing function	Reforms
Revenue raising	Increase public funding to health: expand contributory health insurance; finance to influence health and healthy behaviours using sin taxes.
Pooling	Merge different health coverage programmes; establish or expand Compulsory health insurance; harmonize different funding sources; cross-subsidisation.
Purchasing	Prioritise primary health care services; provider selection; performance-based financing/results-based financing.
Public finance management	Programme-based budget; direct facility financing.

### Revenue raising

Countries’ survey responses and national health financing strategy documents indicate that they are adopting various revenue-raising reforms. These include increasing government budget allocation to health, expanding contributory health insurance schemes, and implementing health taxes.

### Increase in government budget allocation to health

All 18 countries with a current and endorsed health financing strategy emphasised the need to increase public funding to health. Nine countries (Chad, Comoros, Côte d'Ivoire, Kenya, Malawi, Mali, Senegal, Sierra Leone, and The Gambia) specifically aimed to raise the government health budget to 15% of their overall budget, either as a short- or a long-term goal to meet the Abuja target. To achieve this target, countries proposed using evidence-based advocacy and dialogue to engage their ministries of finance or parliaments in discussions to increase government funding for health.

If countries fulfil their commitment to increasing public health spending, it could increase progress towards UHC goals and improve overall health status. Increasing fiscal space in the region is mainly through public health spending [22,23]. Various studies using different data sources have shown that public health spending reduces out-of-pocket spending and increases service coverage [24-26], as well as improves health outcomes [27,28]. While economic growth is necessary for increasing public health spending, it is not sufficient on its own for substantial increase in health spending [29]. Strong political commitment to prioritising public health spending is equally essential to achieving these improvements.

### Expand contributory health insurance schemes

The countries in the region currently have various types of pre-payment schemes (Figure S2 in the **Online Supplementary Document**): approximately 50% of them, mainly from West Africa (Figure S3 in the **Online Supplementary Document**), have compulsory health insurance as the predominant pre-payment scheme, primarily through contributory national health insurance (NHI) or formal sector employee schemes. About one-third of the countries have voluntary schemes such as community-based health insurance/*Mutuelles de Santé* and private health insurance.

Twenty-six of the countries (60%) are currently undergoing or planning reforms related to contributory health insurance schemes (Figure S4 in the **Online Supplementary Document**). Specifically, four countries (Ghana, Rwanda, Senegal, and Zambia) are taking steps to optimise their established schemes. Ghana and Rwanda have proposed refining the premiums paid by the informal sector and increasing enrolment, alongside other improvements in revenue sources, to ensure sustainable financing. Zambia, on the other hand, has transferred the administration of its NHI from its Ministry of Labour back to its Ministry of Health.

**Figure 1:**
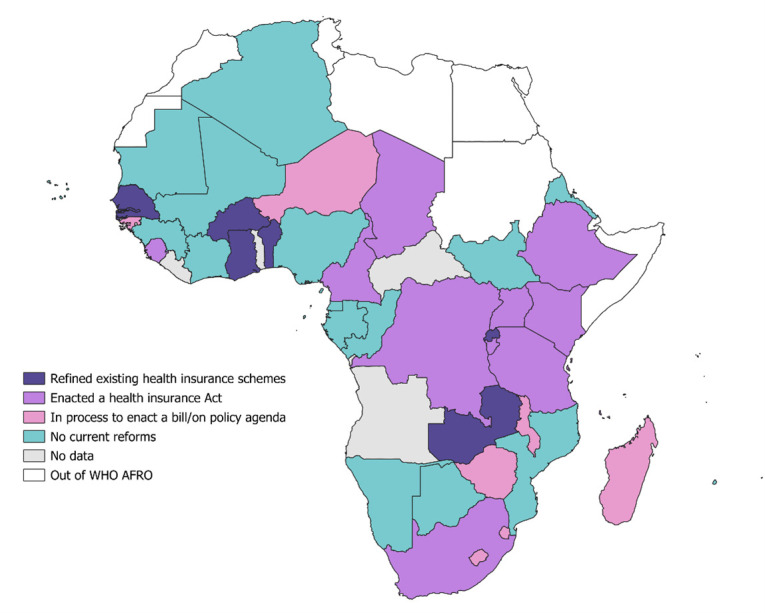
Reforms related to contributory health insurance schemes in the WHO African Region.

Fourteen countries, mainly from East and Southern Africa sub-regions (**Figure 1**), have enacted laws to establish health insurance schemes, with all planning to establish NHI, except for one which is planning on SHI for its formal sector employees. Eight countries, including Malawi and Zimbabwe, have it on their policy agenda to assess the feasibility of establishing a contributory health insurance scheme. Previously, Malawi, according to its Health Financing Strategy 2023–30, decided against establishing an NHI based on the results of a feasibility study [30]. Instead, the country had proposed encouraging participation in private health insurance as an optional coverage for those who may choose not to use public healthcare services.

Evidence on the ability of contributory health insurance schemes to raise revenue is limited in the region. Studies in similar settings have shown that countries face challenges in raising adequate revenue from contributory health insurance such as SHI due to issues such as evasion or incomplete payment of contributions [31]. Furthermore, the revenues generated from contributory health insurance in the region are often regressive. This is particularly true for those in the informal sector, as premiums are typically charged at a flat rate, without accounting for income levels [32]. This can place a disproportionate financial burden on low-income individuals, making financing of such schemes less equitable overall.

#### Financing to influence health and healthy behaviours

In the survey, only Benin, Burkina Faso, and Guinea reported reforms related to health taxes or subsidies as instruments for influencing health behaviours. However, many countries in the region have already imposed health taxes on harmful goods and environmental pollutants. The most common mechanisms include excise tax on tobacco products (in 43 countries), excise tax on alcoholic beverages (in 41 countries), sugar-sweetened beverages (in 7 countries), and tariff on oil, gas, and minerals tax (in 4 countries) [33]. In the review of the 18 countries with a national health financing strategy, 13 explicitly proposed increasing health (sin) taxes targeting risky behaviours and/or taxes on environmental pollutants. Among those that did not explicitly mention health taxes or subsidies, Ghana noted that increasing health taxes was not a viable option due to the Earmarked Funds Capping and Realignment Bill 2017: this legislation capped earmarked revenue at 25%, which has ultimately affected the revenue of its NHI scheme [34].

### Pooling

**Figure 2:**
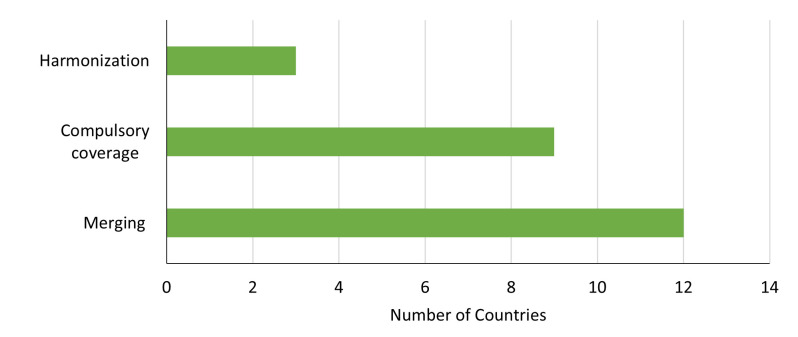
Number of countries with current or planned reforms for pooling. Countries reported more than one type of pooling reform.

Out of the 43 that responded to our survey, 15 (34.88%) have current or planned reforms in their pooling mechanisms, with some countries having multiple reforms (**Figure 2**). Most of these reforms aim to merge pools followed by the introduction of compulsory coverage. A few countries (Côte d’Ivoire, Ethiopia, and Uganda) have planned or ongoing reforms to harmonise their various health coverage arrangements.

#### Merge pools

Several countries are undertaking reforms to merge health financing pools. These include Burkina Faso, Burundi, Cameroon, Democratic Republic of Congo, Gambia, Kenya, Niger, South Africa, Tanzania, Uganda, and Zimbabwe. Kenya has consolidated its various health coverage arrangements into three distinct funds: the Primary Healthcare (PHC) Fund, the SHI Fund, and the Emergency, Chronic, and Critical Illness Fund under the SHI Act. Kenya also integrated its free maternal care policy known as the *Mama Linda* programme into the SHI Fund. Other countries (except for Niger) plan to merge pools by creating NHI systems. Burkina Faso is also transferring the management of its free healthcare policy to its NHI agency to harmonise health coverage programmes. Niger is taking a different approach by planning to merge out-of-pocket payments through voluntary health insurance schemes, such as *Mutuelles de Santé* and Private health Insurance.

#### Compulsory/automatic coverage

All the countries that aim to consolidate or create new schemes also plan to make the revised or new scheme compulsory, except for Niger. Enforcing the compulsory health insurance enrolment may be challenging, particularly due to the large informal sector in the region, which complicates execution. For example, Gabon has an estimated enrolment rate of 40.8% of its compulsory health insurance. After 20 years of its implementation, Ghana’s NHI has achieved only about 80% coverage, with ongoing efforts to include uninsured individuals in the scheme. In Rwanda, high health insurance enrolment has been attributed to its unique political and ideological landscape, which has facilitated the enforcement of compulsory enrolment [35]. However, Rwanda has yet to reach universal coverage, and its health financing strategy proposes to expand the pool size, particularly among young individuals [36]. Algeria, whose compulsory health insurance schemes covers more than 80% of citizens, has less than 35% of its share of employment in the informal economy [37].

#### Harmonisation

Only four countries (Côte d’Ivoire, Ethiopia, Nigeria, and Uganda) reported undertaking reforms to reduce fragmentation through harmonising funding by various health stakeholders. Ethiopia and Uganda are developing mechanisms for a virtual pool of public funds, including external resources, to ensure effective use of resources and alignment of resource allocation to sector priorities. Côte d’Ivoire is creating a national health financing coordination platform to align stakeholders with national priorities, while Nigeria is developing a platform for a sector-wide approach through its Basic Healthcare Provision Fund. To further address fragmentation due to multiple pools, a few countries have proposed reforms to harmonise funding, particularly from external resources. Evidence from Malawi indicates that a sector-wide approach successfully directed investments in cost-effective interventions under the country’s essential health package, leading to increased coverage of essential services despite the package being under-funded [38].

As fragmentation caused by multiple pools from donor funding is a significant gap identified in the literature [39,40], more countries need to continue implementing effective reforms to mitigate this issue. There should be more initiatives to integrate vertical disease and targeted population programmes sponsored by external donors into general government budgets.

#### Cross-subsidisation

None of the countries that responded to the survey indicated any current or planned reforms related to cross-subsidisation. However, in their current health financing strategy, six out of 18 countries (Nigeria, Rwanda, Uganda, Zambia, and Zimbabwe) indicated specific reforms related to improve cross-subsidisation through the creation of risk equalisation mechanisms. Effective risk equalisation could improve equity by reducing incentives to discriminate against sicker or poor individuals and lowering the financial risk for health insurance agencies [41]. However, its implementation could present challenges, particularly in determining how insurance agencies would be compensated and developing the information systems needed to assess risk across populations.

### Purchasing

**Figure 3:**
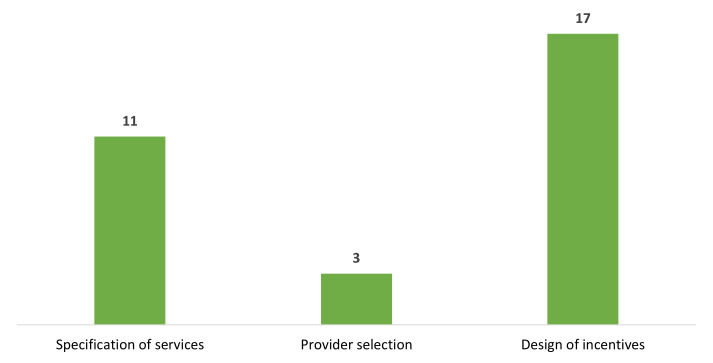
Number of countries with current or planned reforms in purchasing. Three countries had in reforms in more than one core area.

Twenty-four out of the 43 responding countries have current or planned reforms in purchasing, with some having multiple reforms in the different purchasing arrangements (**Figure 3**). Most of these reforms include the scaling up of provider incentives, followed by the selection of services. A few countries have reforms in provider selection.

#### Prioritisation of PHC services

Three countries (Ghana, Lesotho, and Madagascar) are increasingly prioritising purchase of PHC services in their current or planned reforms (**Figure 3**). In Ghana, PHC constitutes only 21% of its NHI expenditure compared to 79% spent in secondary and tertiary levels of care, motivating the country’s UHC roadmap and health financing strategy to shift funds towards it [34]. Lesotho has developed an essential health package as part of its PHC reforms, while Madagascar is considering mechanisms to effectively finance its PHC.

The remaining countries (Benin, Burkina Faso, Burundi, Democratic Republic of Congo, Côte d’Ivoire, Gambia, Guinea, Niger, and Sierra Leone) are also prioritising essential services, particularly for pregnant women and children, by exempting these groups from user fees or any other co-payments. Some countries, such as Benin, Burkina Faso, and others, had an existing free healthcare policy, but these new reforms seek to expand the benefit packages for these groups and increase free services for other vulnerable populations. To ensure effective service coverage, it is crucial to provide adequate funding and other essential resources based on an explicit costed package of services for vulnerable populations [42].

#### Provider selection

Only three countries (Burkina Faso, Ghana, and Mauritius) are currently undertaking reforms in provider selection. Burkina Faso aims to improve contracting of public and private providers for its NHI by requiring providers to meet at least 60% of accreditation standards. Ghana is strengthening PHC by establishing a network of practice that will deliver preventive and promotive services to their entire enrolled populations. The network of practice implementation will be coordinated with the National Health Insurance Agency and Health Facility Regulatory Agency to ensure harmonisation of accreditation process, payment contracts, and processes [34]. Mauritius is adopting a social contracting approach to contract social organisations to provide HIV services.

#### Design of incentives

Of the 17 countries aiming to improve the design of provider incentives (**Figure 3**), 12 plan to scale up performance-based financing to increase access to quality of care and promote equity in pursuit of UHC. Most of the countries, such as Burundi, Cameroon, Côte d’Ivoire, Lesotho, Malawi, Mali, Senegal, and Uganda, had previously implemented or are currently implementing performance-based financing pilot projects primarily supported by donors with varying designs/approaches. The effects of these PBF piloted projects have been mixed. In the Democratic Republic of Congo, PBF had positive, though moderate impacts on the use of maternal health services, particularly among poorer women [43]. From the health system perspective PBF also led to greater availability of medical supplies, and increased providers’ financial autonomy of funds and enhanced use of information systems to monitor performance. In Burundi, Lesotho, Senegal, Zambia, and Zimbabwe, a difference-in-difference analysis conducted using demographic health surveys reported no impact of performance-based financing on neonatal outcomes and showed limited and variable effects on facility delivery, caesarean-sections, and antenatal care visits and its quality [44]. Although performance-based financing has the potential to steer countries towards strategic purchasing, its effects have been limited in the region due to creating parallel structures with little integration with government health budgets and public finance management systems [45].

Other countries have proposed a blended payment model for various levels of the health system. Currently, Ghana is reforming its payment system in health centres, as they receive limited funding from the NHI scheme compared to other levels of care. It is considering optimising its NHI scheme’s provider payment mechanisms to purchase PHC services and to shift funds to strengthen PHC for UHC through its network of practice. The country is also proposing differentiating frontline PHC services and outpatient speciality services by having distinct benefit packages and payment methods for the two services at health centres. Ethiopia, Madagascar, South Africa, and Rwanda proposed a mixed provider payment including capitation at PHC.

Evidence on the effectiveness of blended payment models in the WHO African Region is limited. In Ghana, studies reported that the shift from the Ghana Diagnostic-Related Groups to capitation in a pilot region in 2012 successfully reduced utilisation of care and claims expenditure [46]. However, the move may have also led to under-provision of care. Challenges such as inadequate public education, policy incompatibility with the health system context and difficulties with assigning beneficiaries to providers led to the suspension of capitation in 2018.

### Public finance management

Ten out of 43 countries (23.26%) have current or planned reforms in public finance management (Figure S4 in the **Online Supplementary Document**). Most countries (8 out of 10) have current or planned reforms in budget formulation to transition to programme-based budgeting, while two have proposed to decentralise public finance management systems.

### Programme-based budgeting

The shift to programme-based budgeting presents an opportunity to address longstanding weaknesses in budget formulation and effectively align health budgets with sector priorities [47]. Countries undertaking these reforms are Algeria, Burkina Faso, Ethiopia, Gabon, Guinea, Mauritania, Namibia, and Uganda. A previous study showed that 18 of 41 countries (44%) in the region, including Burkina Faso, Gabon, and Uganda, had introduced some forms of programme classification in their 2017 health budgets, but budget execution continued to be processed by inputs limiting linkages of budget allocation to sector priorities [21]. Experiences from these countries and others show that introducing programme-based budgeting in weak accountability contexts can create complexities and reduce accountability for results. Programme-based budgeting also needs to be accompanied by additional initiatives to build the capacity of health managers and enable the management and use of funds in a flexible manner, particularly at health facility levels [21,48]. Among the eight countries with reforms to shift to programme-based budgeting, Burkina Faso and Uganda have proposed to increase flexibility to shift funds between budget lines and cost centres.

### Direct health facility financing

Two countries, Malawi and Tanzania, have current reforms related to direct health facility financing. Direct health facility financing refers to health facilities receiving funds directly into their bank accounts from various sources, including government general revenue, to purchase health services through output-based provider payment mechanisms [49]. There are three key principles guiding the implementation of direct facility financing: financial autonomy, purchasing health services through output-based provider payment, and effective facility financial management [49]. Financial autonomy allows health facilities to receive, manage, and account for funds. Output-based provider payment ensures that payment to providers aligns with prioritised services. Effective facility financial management enables health facilities to manage the funds they receive and properly account for them. Having an effective facility management requires the integration of good facility accounting and financial reporting systems and health information systems to improve monitoring and accountability of resources used for service provision [49].

Tanzania introduced direct health facility financing in 2017 to improve service delivery and enhance governance and accountability at the PHC level [50]. Prior to this reform, district authorities managed and controlled funds for PHC facilities, leading to delays in funds disbursement and planning, which negatively impacted service delivery [50]. Evidence indicates that direct facility financing in Tanzania increased the availability of health commodities in PHC facilities [51,52]. However, the financial management system of the programme has faced challenges with poor internet connection and limited financial management capacity among health workers [52,53]. To address these issues, the current reform aims to strengthen governance systems at the district level by decentralising planning, budgeting, monitoring, and evaluation at the health facility level through the establishment of district health facility funds. Further process and outcome evaluations of district facility financing in Tanzania are needed to guide implementation and for other countries to benefit from lessons learned.

## DISCUSSION

Countries in the WHO African region have embarked on different health financing reforms, particularly to expand contributory health insurance schemes and performance-based financing. We discuss below the implications of these reforms and the actions countries need to consider for progressing towards UHC.

### Increase government spending through government health budgets or contributory health insurance schemes

Overall, the share of compulsory health insurance in current health expenditure is low across the region. This is likely due to a combination of factors, such as the large informal sector, accounting for an average of about 80% of the region’s economy, and high unemployment rates that present significant challenges for resource mobilisation and determining optimal contribution rates [23]. These challenges are exacerbated by the limited availability of accurate income data for the sector in many countries. If countries’ goal of establishing contributory health insurance through a national health insurance scheme is to generate additional revenue due to stagnant government spending on health – reflecting the region’s growing affinity for such schemes – they must carefully consider the funding sources required for effective implementation. Even after more than two decades of implementation, Gabon, Ghana, and Rwanda continue to face difficulties in effectively collecting contributions from the informal sector. The share of these three countries’ compulsory health insurance contribution to current health expenditure has never exceeded 20% since their inception, with government subsidies being an important source of revenue for their health insurance schemes [54].

Countries such as Cabo Verde and Algeria, with high UHC service coverage and more than 10% of contribution from compulsory health insurance to their current health expenditure, also have high public spending [5]. Conversely, countries such as Botswana, Eswatini, and Mauritius, which have no contribution of compulsory health insurance to current health expenditure and have high public spending, likewise have a high UHC service coverage index [5]. Furthermore, studies have shown that public spending is associated with financial protection [24]. This underscores the importance of public spending on increasing service access and improving financial protection, while highlighting that having health insurance alone may not be sufficient to make progress towards UHC goals. Governments will need to consider increasing their contributions, whether through their health insurance schemes or government budgets, to increase effective service coverage and improve financial protection.

### Harmonise new health coverage schemes with existing schemes to reduce fragmentation

Countries introducing new schemes, such as free healthcare programmes or contributory health insurance schemes, must carefully consider how to harmonise them with existing schemes to avoid fragmentation. Experience from countries such as Ghana suggests that merging pools through NHI can reduce fragmentation and improve the redistributive capacity of prepaid funds [54]. By consolidating various prepaid sources into one fund, an NHI can offer uniform benefit packages, provider payment methods, and other contracting arrangements such as service delivery contracting. However, its establishment often introduces another purchasing agency and could lead to further fragmentation if not carefully managed. To reduce this, actions must be taken to ensure the NHI’s benefit package, provider payments and rates, and contracting mechanisms are harmonised with existing health coverage arrangements, such as the government health budget and targeted population programmes. This harmonisation is essential to ensure that the NHI system integrates effectively with existing health coverage programmes and does not inadvertently create additional barriers or complexities for beneficiaries.

Furthermore, countries’ free health programmes and similar schemes must ensure that benefit entitlements are not duplicated. For example, there should be clear guidance on how free maternal health services will operate when patients are enrolled in SHI schemes that cover maternal healthcare within the benefit package. This raises questions about the scheme and who bears the financial responsibility in such scenarios. Kenya, more than a decade after introducing its free maternal care policy, has faced challenges in aligning this policy with other health coverage programmes. Initially managed by its Ministry of Health, the programme was later transferred to the NHI authority, where its benefit package was enhanced and rebranded as the *Linda Mama* Programme [55]. However, providers have struggled with delayed reimbursements and low payment rates from the NHI Fund while beneficiaries continue to lack access to some of the services under the benefit package, often leading to out-of-pocket payments. Following the merging of schemes under the SHI Act, the *Linda Mama* Programme was incorporated into the SHI Fund in 2024 after public debates regarding its future. It will be important to assess the incorporation of the *Linda Mama* Programme in the health insurance on efficiency and service coverage.

### Manage political economy factors that impede reforms’ goals

Introducing health financing reforms is inherently a political process involving multiple stakeholders with varying levels of power and competing interests [56]. Implementing new revenue-raising schemes or reducing fragmentation across different pools could jeopardise the interests of public and private providers, private sector organisations, donors, and beneficiaries [57]. Therefore, effectively addressing the political economy factors is pertinent to prevent reforms from being blocked or steered away from their initial objectives.

As some countries in the region pursue the implementation of national health insurance schemes, managing political economy factors becomes more crucial to avoid delays or deviation from intended goals. For example, in Zambia, the government needed to negotiate with trade unions to lower the SHI contribution initially derived from actuary analyses to pass its national health insurance bill [58]. Similarly, Kenya’s experience of integrating its free maternal care policy under the SHI Act shows the importance of political economy factors when seeking to address fragmentation from multiple health coverage programmes. Public scrutiny over the failure to explicitly include the free maternal care policy in the new SHI Act led the government to decide to fully incorporate the maternal care services into the country’s SHI Fund. In Ghana, the rollout of capitation in one region faced significant political economy challenges from the public and providers. These challenges stemmed from its framing as a cost containment strategy and as a political tactic against the opposition stronghold in the pilot region. These examples highlight the importance of framing reforms appropriately and engaging diverse stakeholders early in the process to ensure smooth implementation.

### Align reforms in public finance management systems with provider payment reforms

More countries will need to make changes in their PFM systems to align with reforms in provider-payment mechanisms. The results from the survey showed that many countries in the region are shifting towards strategic purchasing through performance-based financing, but few are undertaking reforms in PFM to support this incentive structure. Studies have identified limited financial autonomy in health facilities and input-based budgeting as significant challenges in the region. Line-item budgeting results in unrealistic budget proposals and misalignment with sector needs. It also restricts flexible spending, making it difficult for the health sector to address emerging needs. Shifting to budget systems that align with new provider-payment mechanisms through SHI or performance-based financing programmes could create the appropriate incentives to improve efficiency and service delivery.

## CONCLUSIONS

Adopting and implementing evidence-based, context-specific health financing reforms is crucial for advancing progress towards UHC. Countries’ survey responses and national health financing strategies indicate that countries have committed to establishing various health financing reforms, particularly in the areas of revenue raising and purchasing functions. Reforms such as performance-based financing, direct facility financing, and prioritisation of PHC services show potential for improving the quality of care and equity in service coverage. However, ensuring these reforms are implemented within appropriate public finance management structures and avoiding further fragmentation will enhance their effectiveness.

Other reforms, such as compulsory health insurance schemes, have the potential to reduce fragmentation by merging various health coverage programmes. However, challenges remain in population coverage, limited ability to raise substantial revenue for health, and weak capacity to expand service coverage. Despite the limited evidence on the effectiveness of contributory health insurance in lower-income contexts for achieving UHC goals, demand for this health financing mechanism is growing in the region. Countries’ commitment to increase public health spending provides a foundation for progress, but stakeholders must support them countries in addressing the political economy factors that drive the preference for contributory health insurance and promote the most effective reforms to achieve UHC.

## Additional material


Online Supplementary Document
