# Supplementary material for: Countdown to 2030: overview of current and planned health financing reforms for universal health coverage in the WHO African Region
Source: J Glob Health. 2025 Aug 8;15:04233. doi: 10.7189/jogh.15.04233 (PMC12333570; doi:10.7189/jogh.15.04233)
Supplement: Online Supplementary Document [file jogh-15-04233-s001.pdf]

Supplement to: Osei Afriyie D, Karenzi Muhongerwa D, Nabyonga-Orem J, Chukwujekwu O. Countdown to 2030: overview of current and planned health financing reforms for universal health coverage in the WHO African Region. J Glob Health. 2025;15:04233.

Figure S1: WHO's Health Financing for UHC framework. Source: Adapted from Kutzin, 2013 [4].

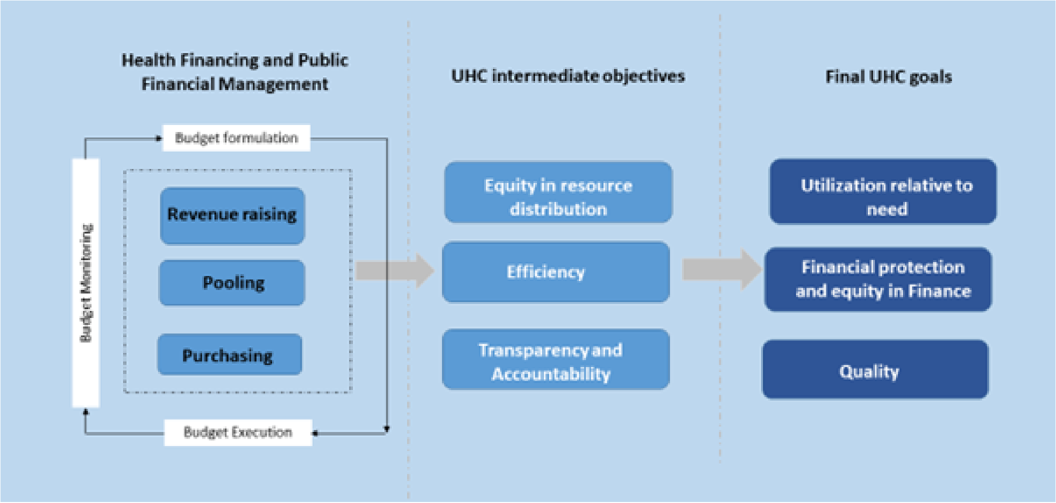

Figure S2: Percentage of countries with established prepayment mechanisms in the African Region in 2024. Predominant pre-payment scheme in countries.

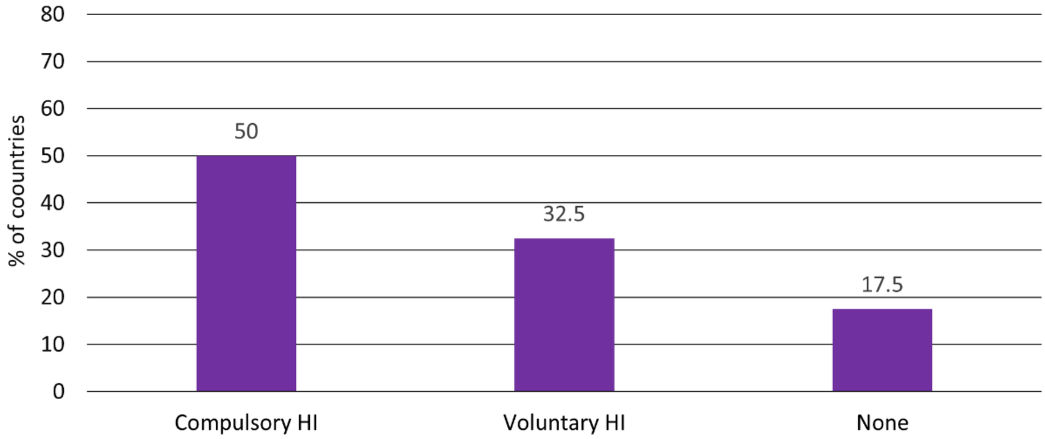

Figure S3: Predominant Health Insurance Schemes in the WHO African Region (WHO AFRO)

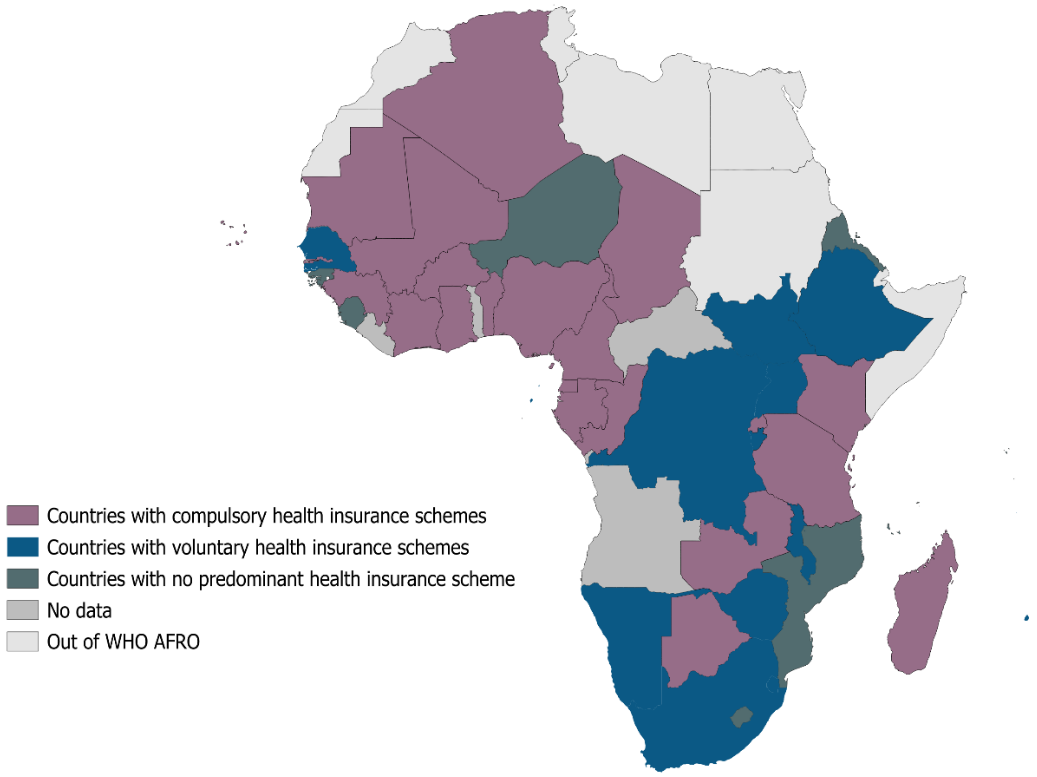

Figure S4: Number of countries with current or planned reforms for contributory health insurance schemes in 2024

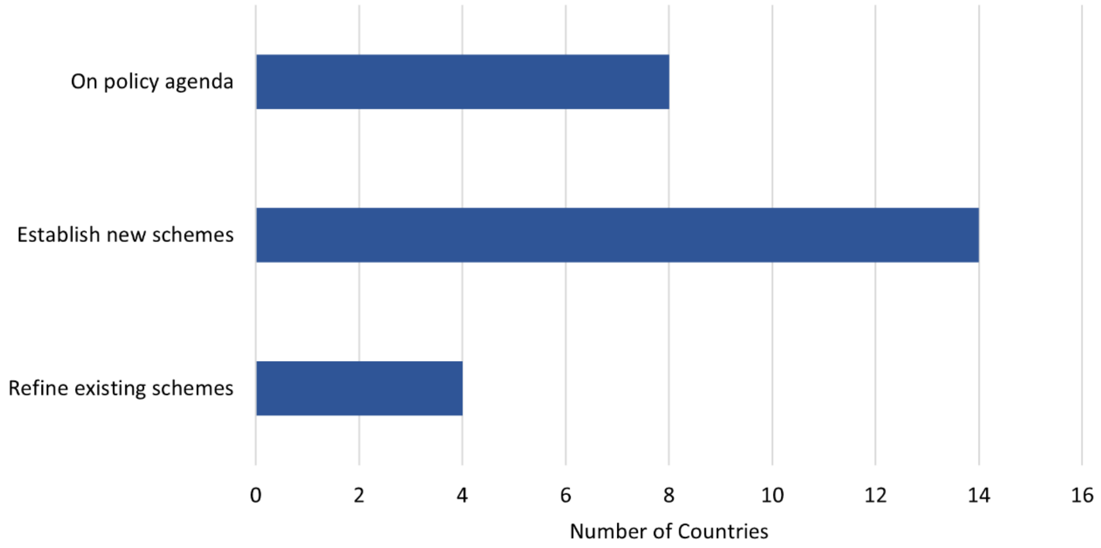

Supplementary Table 1: English Form of Survey assessing countries' current and planned health financing strategies and assessments

Questions	Responses
1. Have you ever had a health financing strategy?	Yes or No

2.	Do you have a health financing strategy?	Yes or No
3.	If yes, indicate the period covered by the health financing strategy	
4.	If No, is there a plan to develop a health financing strategy? Please indicate any bottleneck that the country might have.	
5.	For countries that have health financing strategy, please provide a copy or link to the plan	
6.	For countries planning to develop/review Health Financing Strategy, please indicate the period	Yes or No
7.	Has the country conducted health financing systems assessment recently (including assessments not supported by WHO)?	Yes or No
8.	If Yes, please indicate the type of assessment used (i.e: HFPM or others)	
9.	For countries that have assessed the health financing systems, please provide a copy or link to the reports	
10.	For countries planning to conduct health financing systems assessment, please indicate the period	
11.	Does your country have any form of pre-payment systems for healthcare services?	Yes or No
12.	If yes, what types of pre-payment systems are in place? (national health insurance, health insurance covering a specific group of the population)	
13.	Are these pre-payment systems mandatory or voluntary? Please specify for each pre-payment scheme.	
14.	How would you describe the current stage of implementation for the pre-payment systems in your country?	a) Not started<br>b) Law enacted<br>c) Scheme Management structure(s) in place<br>d) Scheme Management structure(s) operational
15.	Please list any other ongoing Health Financing Reforms in your country, including but not limited to PFM, Strategic Purchasing, Prepayment /Social Protection/Health Insurance	
16.	Please list any planned (2024/25) Health Financing Reforms, including but not limited to PFM, Strategic Purchasing, Prepayment /Social Protection/Health Insurance	
17.	For countries planning to develop health financing reforms, would this be initiated within a given period e.g. this year versus next year?	

Supplementary Table 2: Countries with current validated health financing strategy in 2024

Country	Period
Comoros	2019-2030
Ethiopia	2022-2031
Kenya	2020-2030
Malawi	2023-2030
Rwanda	2018-2024
South Africa	2020/21 - 2024/25
Tanzania	2015-2026
Uganda	2015/16 - 2024/25
Zambia	2017-2027
Burkina Faso	2018-2030
Côte d’Ivoire	2015-date
Gambia	2019 - 2024
Ghana	2023-2030
Mali	2014-2023
Niger	2023-2030
Nigeria	2017-date
Sierra Leone	2021-2025
Chad	2021-2030

Summary of countries' current and planned reforms in 2025.

Country	Revenue Raising	Pooling	Purchasing	Public finance management
**Algeria**	None		None	Ongoing project for the implementation of program-based budgeting for healthcare
**Angola**				
**Benin**	•Establish NHI<br>•Innovative financing	Mutuelles de santé, Private health insurance	Free healthcare initiative for caesarean section, pregnant women, and children under five	Non
**Botswana**	None	None	None	
**Burkina Faso**	Establish Universal Health Insurance	•Transfer of the management of the current system of free health care to the National Universal Health Insurance Fund (CNAMU).<br>•Harmonisation of practices: benefits package and pricing system<br>•Operationalisation of Universal Health Insurance	•Increase the basket of free care: by adding PLHIV, the elderly, chronic diseases (cancer, heart disease) and SAMU.<br>•Change of payment method: share of medicines (highlighting the share of medicines in invoices).<br>•Strengthening of contracting: public and private (at least 60% quality)	•Programme based budgeting<br>•Reduce rigidity to increase flexibility between budget lines
**Burundi**	Establish Compulsory HI	Public health insurance, Private health insurance	Free fees, Performance-based financing	None

**Cameroon**	Establish CHI	Compulsory Health Insurance	Strategic purchasing/Provider payments	None
**Cape Verde**	None	None	None	None
**Central African Republic**			None	None
**Chad**	None	None	None	None
**Comoros**	Establish NHI	National Health Insurance	Provider payments	None
**Congo**	None	None	None	None
**Congo DRC**	Establish NHI	Compulsory Health Insurance (enacted by law)	Free healthcare initiative for pregnant women and new-borns	None
**Cote d'Ivoire**	Transition to domestic financing with the end of eligibility for funding from global initiatives (GAVI, Global Fund, etc.)	Creation of a national platform for coordinating health financing	Free services for pregnant women and children, PBF	None
**Eritrea**	None	None	None	None
**Eswatini**	Ongoing discussion to establish NHI	Ongoing discussion to establish NHI	None	None
**Ethiopia**	None	Virtual pooling of funds	Results based financing, Blended Provider payments	Programme-based budgeting
**Gabon**	None	None	None	Programme-based budgeting
**Gambia**	Establish NHI	Establish NHI	Bamako Initiative, Results Based Financing	None

**Ghana**	Addressing NHIS revenue issues including SSNIT /Capping - Ongoing advocacy leading to a decrease in the capping percentage from 25% to 17 %	None	•Strengthening purchase of PHC services-Establishment of NOPs currently ongoing<br>•Differentiate front-line PHC services, outpatient speciality services and the payment systems to providers or management entities that deliver them.	None
**Guinea**	None	None	•Social development Funds for Indigents (FDSI)<br>•Performance-based Financing	Programme-based budgeting
**Guinea Bissau**	On policy agenda for contributory health insurance		None	None
**Equatorial Guinea**	None	None	None	None
**Kenya**	Establish 3 Funds	Establish 3 Funds	Provider Payment systems lined to Performance and Quality, QOC Bill	None
**Lesotho**	Establish CHI in 2025	Establish CHI in 2025	•Prioritization of essential services<br>•Performance-based financing	None
**Liberia**				
**Madagascar**	On policy agenda for mutuelles de santé	On policy agenda for mutuelles de santé	Capitation and blended payments	None
**Malawi**	On policy agenda for contributory health insurance	On policy agenda for contributory health insurance	Performance-based financing	Direct health facility financing
**Mali**	Innovative financing	None	Performance based financing	None

**Mauritania**	None	None	Performance based financing	Programme-based budgeting
**Mauritius**	None	None	• Social contracting of HIV programme<br>• Recently, MOUs were developed as part of a Public Private Partnership initiative whereby for high tech medical services the public sector would outsource with the private clinics rather than providing direct assistance for patients who require treatment abroad	None
**Mozambique**	None	None	None	None
**Namibia**	None	Program based budgeting	None	Programme-based budgeting
**Niger**	Establish Mutuelles de santé	Establish mutuelles de santé, Private health insurance	Targeted free healthcare system	None
**Nigeria**	None	Basic Healthcare Provision Program using the Sector Wide Approach (SWAp)	None	None
**Rwanda**	Refine CBHI for sustainability	Refine CBHI for sustainability	Provider Payment Mechanism (Capitation for PHC)	None
**Sao Tome and Principe**	None	None	None	None
**Senegal**	Extension of Universal Health Coverage (CMU)	Extension of Couverture Maladie Universelle (CMU)<br>Change of the management structure of	Performance based financing	None

		CMU to the National Universal Health Coverage Agency (CSU)		
**Seychelles**	Facilitate voluntary PHI	None	None	None
**Sierra Leone**	Establish Sierra Leone Social Health Insurance (SLeSHI)	Development of the UHC Act and operationalisation of SLeSHI by November	Reform/Review of the free care policy	None
**South Africa**	Establish NHI	Establish NHI	Capitation for Primary care level, DRGs for Strategic purchasing and provider payment for secondary and tertiary care level, accreditation etc	None
**South Sudan**	None	None	None	None
**Tanzania**	Establish NHI	Establish NHI	None	Direct health facility financing
**Togo**				
**Uganda**	Establish NHI	Virtual pooling of funds, Establish NHI	Results Based Financing	None
**Zambia**	Refine scheme-return to MoH	None	None	Programme-based budgeting
**Zimbabwe**	On policy agenda for contributory health insurance	On policy agenda for contributory health insurance	None	None
